# Environmental variation associated with overwintering elicits marked metabolic plasticity in a temperate salmonid, *Salvelinus fontinalis*

**DOI:** 10.1242/jeb.246743

**Published:** 2024-02-12

**Authors:** Ella K. Middleton, Matthew J. H. Gilbert, Thomas Landry, Simon G. Lamarre, Ben Speers-Roesch

**Affiliations:** ^1^ Department of Biological Sciences, University of New Brunswick, Saint John, Canada, E2K 5E2; ^2^ Département de Biologie, Université de Moncton, Moncton, Canada, E1A 3E9

**Keywords:** Brook char, Thermal acclimation, Energy expenditure, Oxygen uptake, Protein synthesis, Food deprivation, Winter

## Abstract

Poleward winters commonly expose animals, including fish, to frigid temperatures and low food availability. Fishes that remain active over winter must therefore balance trade-offs between conserving energy and maintaining physiological performance in the cold, yet the extent and underlying mechanisms of these trade-offs are not well understood. We investigated the metabolic plasticity of brook char (*Salvelinus fontinalis*), a temperate salmonid, from the biochemical to whole-animal level in response to cold and food deprivation. Acute cooling (1°C day^−1^) from 14°C to 2°C had no effect on food consumption but reduced activity by 77%. We then assessed metabolic performance and demand over 90 days with exposure to warm (8°C) or cold winter (2°C) temperatures while fish were fed or starved. Resting metabolic rate (RMR) decreased substantially during initial cooling from 8°C to 2°C (*Q*_10_=4.2–4.5) but brook char exhibited remarkable thermal compensation during acclimation (*Q*_10_=1.4–1.6). Conversely, RMR was substantially lower (40–48%) in starved fish, conserving energy. Thus, the absolute magnitude of thermal plasticity may be masked or modified under food restriction. This reduction in RMR was associated with atrophy and decreases in *in vivo* protein synthesis rates, primarily in non-essential tissues. Remarkably, food deprivation had no effect on maximum oxygen uptake rates and thus aerobic capacity, supporting the notion that metabolic capacity can be decoupled from RMR in certain contexts. Overall, our study highlights the multi-faceted energetic flexibility of *Salvelinus* spp. that likely contributes to their success in harsh and variable environments and may be emblematic of winter-active fishes more broadly.

## Teaser

**Summary:** Food deprivation and frigid temperatures, common winter challenges, elicit tissue-specific energy conservation mechanisms and thermal compensation in brook char. Despite lower resting metabolism during starvation, aerobic metabolic capacity was maintained.

## INTRODUCTION

Temperate winters are commonly characterized by frigid temperatures and low food availability, which pose distinct energetic challenges for ectotherms (Fernandes and McMeans, 2019; Shuter et al., 2012). The impact of food deprivation on survival and reproductive success depends on existing endogenous energy stores, the ability to acquire exogenous energy despite reduced availability, and the rate of energy expenditure (Hurst, 2007; Secor and Carey, 2016). Frigid temperatures can exacerbate or alleviate winter energy limitations. Specifically, cold can constrain physiological functionality, in part, by slowing biochemical reaction rates (Schulte, 2015), which can impair feeding and digestion (Fernandes and McMeans, 2019). However, this same passive slowing effect markedly reduces energy expenditure, with whole-animal metabolic rate generally decreasing by >50% for every 10°C of cooling (*Q*_10_∼2–3; Clarke, 2017; Clarke and Johnston, 1999).

In response to winter cold, some fish become dormant or lethargic, feeding minimally if at all (Reeve et al., 2022), whereas others, including most salmonids, remain relatively active (Auer et al., 2020; Blanchfield et al., 2009; Cunjak and Power, 1987; Fraser et al., 1993). Many winter-active fishes upregulate metabolic processes to partially compensate for constraints imposed by the cold (i.e. thermal compensation via acclimation). However, this compensation raises whole-animal resting metabolic rate, thereby counteracting beneficial energy savings of cooling (Guderley, 1990; Norin and Speers-Roesch, 2021). Although intuitive, the extent of this trade-off is poorly understood and the combined energetic effects of food deprivation and cold have rarely been examined in fishes, despite being common co-occurring winter challenges (Huey and Buckley, 2022).

Metabolic flexibility could allow fishes to balance their energy expenditure over winter in a context-dependent manner (Auer et al., 2016b; O'Connor et al., 2000; Reeve et al., 2022; Speers-Roesch et al., 2018; Van Leeuwen et al., 2012). For instance, when faced with food deprivation, fish could conserve energy by blunting metabolic thermal compensation. A lack of compensation of resting metabolic rate has been previously observed in winter-dormant and lethargic fishes (Healy et al., 2017; Reeve et al., 2022), but a mechanistic basis is unclear. Alternatively, metabolic thermal compensation could be retained but additional energy conservation mechanisms could be employed; beyond reducing activity, fish could actively downregulate specific costly physiological functions (e.g. protein synthesis, mitochondrial abundance; Salin et al., 2018) in non-critical tissues (e.g. digestive system), thereby reducing whole-animal energy expenditure while maintaining the functionality of critical tissues. Indeed, a primary way that other fasting ectotherms and hibernating mammals reduce energetic demands in food-poor environments is through such targeted reductions in resting metabolic costs (Auer et al., 2016b; Naya et al., 2009; Tøien et al., 2011; Van Leeuwen et al., 2012). However, this downregulation could be accompanied by a reduction in maximum performance (e.g. digestive capacity or maximum metabolic rate, MMR) (Bennett and Ruben, 1979); higher resting metabolic rates may be associated with greater maximal metabolic performance, as it costs energy to maintain the machinery supporting elevated metabolic performance (Auer et al., 2015; Guderley, 2004; Monternier et al., 2014) (but see Auer et al., 2016a,b; Barceló et al., 2017; Koteja, 1987; Salin et al., 2016; Zeng et al., 2018). In contrast, in more food-rich environments, the maintenance and thermal compensation of digestive and aerobic capacity may justify any additional cost (i.e. elevated resting metabolic rate). Still, we have limited insight into how winter-active fishes balance the trade-offs between the potential need for energy conservation with the cost of maintaining an active, high-capacity phenotype over winter. This knowledge gap is of particular concern as winter conditions are rapidly changing, which may impact the efficacy of fishes' overwintering strategies (Morash et al., 2021).

To address this knowledge gap, we studied brook char, a temperate salmonid native to northeastern North America (MacCrimmon and Campbell, 1969). Brook char, like most salmonids, can remain active and feeding in the winter (Cunjak and Power, 1987; Cunjak et al., 1987) and can experience food limitation (Spares et al., 2014), making them ideal to study energetic trade-offs associated with overwintering. First, to determine the effect of cooling on voluntary feeding and activity, which are key energetic traits, brook char were gradually cooled from 14°C to 2°C while being fed daily and monitored continuously. In our second experiment, we exposed brook char to two ecologically relevant winter water temperatures (8°C and 2°C) for up to 90 days while either fed or starved. To understand the metabolic and energetic impacts of these conditions, we assessed resting and maximum oxygen uptake, tissue-specific energy demands (*in vivo* protein synthesis rate and organ sizes) and endogenous energy storage. Resting metabolic rate was assessed at four time points over 90 days to reveal the temporal dynamics of plasticity in response to cold and food deprivation, a valuable approach to study acclimation that is rarely employed (Havird et al., 2020; Somero, 2015). Given the well-documented environmental plasticity of *Salvelinus* fishes (Armstrong and Bond, 2013; Dutil, 1986; Gilbert and Farrell, 2021; Hutchings, 1996; Muir et al., 2016), we hypothesized that these winter-active fish possess the metabolic flexibility to balance energetic trade-offs of combined variation in temperature and food availability. Accordingly, we predicted that brook char would thermally compensate for prolonged exposure to 2°C resulting in an increase in resting metabolic rate over time, but to a greater extent in fed fish. Furthermore, we predicted that brook char would reduce resting metabolic rate in response to starvation, conserving energy but compromising maximum aerobic performance. Finally, we expected reductions in resting metabolic rate to be largely driven by reductions in energy demand in tissues and processes associated with growth and digestion.

## MATERIALS AND METHODS

All experiments were approved by the Animal Care Committee of the University of New Brunswick (UNB), Saint John, following the Canadian Council on Animal Care standards (UNB 2020-4S-03).

### Experiment 1: the effect of cooling on activity and food consumption of brook char

#### Experimental animals

Juvenile (<1 year) brook char, *Salvelinus fontinalis* (Mitchill 1814), were obtained from a captive breeding population at UNB Fredericton, New Brunswick, Canada, in October 2020. They were held at UNB Saint John in 400 litre circular fibreglass holding tanks supplied with flow-through dechlorinated freshwater (∼15°C) and fed dry pellets (1.8 mm Gemma, Skretting, St Andrews, NB, Canada) to satiety three times per week. During holding and both experiments, fish were always under a winter photoperiod (10 h:14 h light:dark) typical for temperate latitudes; a simulated sunrise and sunset (30 min each) were included in the lighting period to minimize potential effects of sudden lighting changes on behaviour (Ryu et al., 2020).

Brook char used for experiment 1 (*n*=15, length=82.9±5.8 mm, mass=5.30±0.84 g, means±s.d.) were transferred to three 90 litre glass aquaria (five fish per aquarium) 2 weeks before the start of the experiment. These tanks were supplied with recirculated temperature-controlled filtered dechlorinated freshwater (14±0.5°C) and fed dry pellets once per day to satiety.

#### Experimental design

To quantify the behavioural responses of brook char to cooling, we measured spontaneous activity and food consumption during an acute cooling period from 14°C to 2°C (1°C day^−1^), and a subsequent acclimation period of 2 weeks at 2±0.5°C.

Activity was measured as described in Reeve et al. (2022) with minor modifications. Briefly, the housing system consisted of a clear outer acrylic aquarium containing 15 clear plastic arenas (20.2×15.6×9.7 cm), each housing a single fish (*n*=15); each arena received recirculating, filtered, aerated and temperature-controlled freshwater from the external bath. The system was illuminated from below by one near-infrared lamp (940 nm) and fish were continuously video recorded from above with two infrared-sensitive cameras (640×480 pixels, 10–15 frames s^−1^, IDS Imaging, Obersulum, Germany). Fish were allowed 36 h at 14°C to adjust to the arenas with food withheld before the recording of activity and food consumption began. After this adjustment period, the fish were exposed to 14°C for an additional 24 h, following which the system was cooled by 1°C each morning until it reached 2°C. Cooling lasted ∼30 min, during which time feeding counts were performed. Fish were fed a standard ration of dry pellets (0.5% of body mass) each morning and any remaining pellets were counted the following morning to estimate daily food consumption.

### Experiment 2: the effects of prolonged starvation at 8°C or 2°C on brook char energetics and metabolism

#### Experimental animals

The second experiment was conducted with brook char that were obtained in 2018 from the same breeding population as in experiment 1. These fish were reared under the same holding conditions as experiment 1 but were fed larger pellets (23 kJ g^−1^; 40A 4.0 mm BioTrout, Bio-Oregon, WA, USA) prior to the start of the experiment because they were larger. Two weeks before beginning experiment 2, 60 brook char (length=214.3±19.15 mm, mass=137.78±38.36 g, means±s.d.) were distributed equally among four recirculating freshwater systems at 8±0.5°C that served as the treatment systems (see below) once the experiment started. During the 2 weeks before experiment 2 began, all fish were fed dry pellets once per day (∼0.5% average body mass ration per aquarium).

#### Experimental design

Experiment 2 lasted for a total of 96 days and had a full-factorial design. Each of the four treatment systems consisted of a 180 litre sump with biological and solids filtres, and three 90 litre glass aquaria that each housed five fish and contained a cylindrical PVC shelter. The sump was supplied with a constant drip of dechlorinated freshwater (not strong enough to alter water temperatures) to further maintain water quality. Fish in the four acclimation systems were exposed to either 2°C or 8°C (i.e. two systems per temperature) and were either fed daily (∼0.5% body mass ration) or starved (no feed). The temperatures were chosen based on data for winter water temperature throughout the natural range of brook char (Brook Trout Atlas: https://www.tu.org/science/science-engagement/interactive-maps/brook-trout-atlas/; Real-Time Hydrometric Data Map Search: https://wateroffice.ec.gc.ca/map/index_e.html; MacCrimmon and Campbell, 1969; United States Geological Survey National Water Dashboard: https://dashboard.waterdata.usgs.gov/app/nwd/). Given that their range extends to high latitudes and elevations, exposure to stable cold temperatures including 2°C can last for many months. The feeding treatments were applied to aquaria within each treatment system such that fed and starved fish were housed next to each other within the same system at a given temperature. For the fed group, a relatively small ration of 0.5% body mass was chosen to ensure the entirety of the ration was eaten even at 2°C, which standardized food intake between the groups (i.e. fish at 2°C and 8°C ate the same amount). Further, the ration was ecologically relevant in a winter context given that overwintering brook char would often not have regular access to large meals, but was still above maintenance as fed fish at 2°C and 8°C exhibited positive growth (Table 1).

**Table 1.**
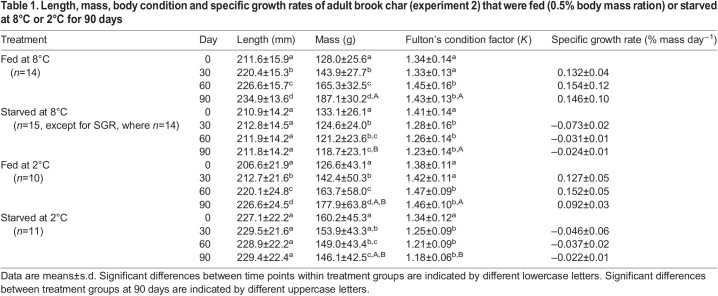
Length, mass, body condition and specific growth rates of adult brook char (experiment 2) that were fed (0.5% body mass ration) or starved at 8°C or 2°C for 90 days

Fish assigned to 2°C treatments were cooled from 8°C to 2°C over ∼10 h (i.e. acutely) immediately prior to the 48 h respirometry measurements that were used to assess the standard rate of oxygen consumption (*Ṁ*_O_2_standard_) as a proxy for standard metabolic rate (see below). We could only perform respirometry on seven fish at the same time and so the start of the 2°C treatments had to be staggered to allow time for respirometry measurements. As such, seven fish from a given housing system were transferred to the respirometers at 8°C and then cooled prior to the start of the measurements. The remaining fish in that housing system were then cooled immediately in the housing system, prior to being transferred to the respirometer. This practical approach meant that the first group of fish in the respirometers could be transferred back to the housing tanks which were now at 2°C, and the next group placed in the respirometers already at 2°C while ensuring all fish tested at 2°C had the same thermal experience prior to the estimation of *Ṁ*_O_2_standard_. ‘Day 0’ for fish assigned to the 2 and 8°C was considered as 24 h after fish had first been in the respirometers at their acclimation temperature. In all cases (fed and starved), food was withheld for a total of 72 h before the point considered ‘day 0’ to ensure fish were in a post absorptive state during the first estimation of *Ṁ*_O_2_standard_. Food was then withdrawn for the remainder of the experimental acclimation period for fish in the starved treatment whereas the daily ration was resumed for those in the fed treatment. Note that on the 35th day of the experiment, a chiller malfunctioned on one of the two 2°C systems and water temperature increased to a maximum of 11.7°C overnight. The following morning temperature was lowered gradually back to 2°C over the course of ∼12 h with no short-term mortality.

#### Oxygen consumption rate analysis and spontaneous activity

Aerobic metabolic rates were estimated using the measurements of oxygen consumption rates (*Ṁ*_O_2__; mg O_2_ kg^−1^ h^−1^) obtained through automated intermittent-closed oxygen respirometry (Svendsen et al., 2016). We used 6.1 litre (34×23×8.7 cm) plastic respirometers (LOCK & LOCK, Seoul, South Korea). Each respirometer was fit with a recirculating pump (Eheim 300) for constant mixing, and the recirculating loops were fit with individual temperature-compensated optodes (FireSting, PyroScience, Aachen, Germany) to measure dissolved oxygen (DO). DO in an empty respirometer was recorded simultaneously to record background changes in DO that were then subtracted from the fish’s recordings. ‘Day 0’ was defined as described above and subsequent time points for respirometry were considered relative to this starting point. Fish were weighed and measured after each respirometry trial, and standard length and body mass measurements were used to calculate Fulton's condition factor (see ‘Data analysis’). Following the first respirometry trial, fish were tagged (Elastomer implant, Northwest Marine Technology Inc., Anacortes, WA, USA) to allow for subsequent identification. At each subsequent measurement, food was withheld from fed fish for 72 h prior to the period from which standard metabolic rate (SMR) was estimated (i.e. 48 h before the start of respirometry measurements) to allow gut evacuation (Chabot et al., 2016a,b; Cutts et al., 2002).

SMR, the basal maintenance cost of living (Chabot et al., 2016a; Norin and Speers-Roesch, 2021), was estimated (*Ṁ*_O_2_standard_) in each fish at four time points during the experiment (0, 30, 60 and 90 days). The flush period was 2 min, and the closed period was 10 min at 8°C and 22 min at 2°C. *Ṁ*_O_2_ _was measured from the slope of the decline in DO content (mg l^−1^) during each closed period, the data for which were extracted using an automated template in Microsoft Excel (v16.62, Microsoft Corp., 2018). At 2°C and 8°C, the first 4 min of each closed period was excluded from the slope calculation. Within the remaining closed period, we calculated *Ṁ*_O_2__ using one 6-min slope at 8°C and two 6-min slopes at 2°C, resulting in *Ṁ*_O_2__ measurements every 12 min on average at both temperatures despite differing flush cycle lengths. Fish remained in the respirometers for ∼48 h for each *Ṁ*_O_2_standard_ trial, with the initial 24 h being considered an adjustment period, and the lowest 20th percentile of all *Ṁ*_O_2__ measurements in the final 24 h was taken as *Ṁ*_O_2_standard_ (Chabot et al., 2016b). *Ṁ*_O_2_standard_ estimates were generated using several methods to assess whether any differences among groups were a result of changes in body mass, fat content or activity. Regardless of which method was used to estimate *Ṁ*_O_2_standard_, the values, patterns of results, and *Q*_10_ values (see Supplementary Materials and Methods) were similar, and our conclusions were identical, indicating that the 20th percentile method was robust for this study.

One individual was removed from *Ṁ*_O_2_standard_ analysis at 90 days owing to a blockage in the recirculating loop that did not allow for accurate oxygen readings, resulting in a sample size of 13 instead of 14 for starved fish at 8°C in the final time point (90 days). The sample sizes for *Ṁ*_O_2_standard_ of fish starved at 2°C increased from 10 at time 0 and 30 days to 11 at 60 and 90 days. This increase was a result of three mortalities between day 0 and 60 that were entirely removed from the dataset, followed by the addition of a spare individual into respirometry measurements. This spare individual was added to help offset the loss in sample size and was already housed in the appropriate treatment tank but had not previously undergone respirometry. In total, seven mortalities occurred (fed at 2°C: 4; starved at 2°C: 3) throughout the experiment, and these fish were removed from all data analysis.

In addition to *Ṁ*_O_2_standard_, the average *Ṁ*_O_2__ (*Ṁ*_O_2_average_) was taken over the final 24 h of each respirometry trial, which incorporated variation in *Ṁ*_O_2__ that occurred largely because of spontaneous activity of fish in the respirometers (Fig. S1). Spontaneous activity of the fish was measured during respirometry trials using near-infrared-sensitive cameras positioned above the respirometers (720×1080 pixels, 30 frames s^−1^, with two attached illuminators, Zosi Technology Ltd, China; and 720×1280 pixels, 30 frames s^−1^, model ELP-USB3MP01H-RL36, Elpcctv, Ailipu Technology Co., Ltd, China). Average and median activity were calculated at each time point (Fig. S2B,C).

Lastly, the maximum ability to uptake oxygen in support of aerobic metabolism was estimated as *Ṁ*_O_2_max_ once, at 90 days, immediately following the final estimate of *Ṁ*_O_2_standard_. Fish were manually chased for 5 min, a standardized measurement period chosen based on studies of *Ṁ*_O_2_max_ in relation to exercise mode and duration (Little et al., 2020; Zhang et al., 2020). Both previous studies demonstrated that the duration of the sampling window used to extract *Ṁ*_O_2_max_ was a more important consideration than the duration of the chase protocol itself. Additionally, field and laboratory studies on a closely related species, the Arctic char, confirmed that fish were at or near exhaustion by 5 min at cold temperatures (Gilbert, 2020; Gilbert et al., 2020). Following the chase, fish were immediately sealed in the respirometers and the flush pumps remained off until the DO fell by ∼2 mg l^−1^ (>80% air saturation); flush pumps were then turned on to allow DO to rise by ∼0.5 mg l^−1^ and then shut off again to fall by ∼0.5 mg l^−1^, at which time the respirometers were returned to their regular automated flush cycles. If DO levels fell below ∼80% air saturation, respirometers were again manually flushed to increase the DO in the chamber. This manual intermittent procedure maximized the duration of oxygen consumption recordings immediately following chasing to improve the accuracy of *Ṁ*_O_2_max_ estimates. Fish were then left to recover within the respirometers for at least 12 h before being returned to their respective acclimation systems. *Ṁ*_O_2_max_ was taken as the steepest DO slope over 90 s, which was detected using an iterative algorithm applied over all DO recordings following the chase event as previously described (Zhang et al., 2018). The absolute aerobic scope (AAS) of each fish was calculated as the difference between its *Ṁ*_O_2_standard_ and *Ṁ*_O_2_max_.

#### Tissue collection and tissue energetics analyses

Fish were chemically euthanized (300 mg l^−1^ MS-222 and 600 mg l^−1^ sodium bicarbonate, Sigma-Aldrich, St Louis, MO, USA) 3 days following the final respirometry measurements. Blood was immediately extracted via caudal puncture and tested in duplicate for glucose (Accu-Chek, Roche Diabetes Care, Basel, Switzerland) and haematocrit (centrifuged 5 min at 5000 ***g***; Sorvall Legend 17 Microcentrifuge, Thermo Fisher Scientific, Waltham, MA, USA). Body fat (%) was measured using a fish fat meter (FFM-692, Distell, Fauldhouse, UK). Tissue samples (ventricle, liver, gut and white muscle) were dissected, sexed (30 females, 20 males; see dataset at https://figshare.com/s/ec15fb4a979c89aea319), weighed and immediately frozen in liquid nitrogen. Tissue and plasma samples were stored at −80°C until analysis (4–16 months for tissues, ∼19 months for plasma). The remaining carcasses were frozen at −20°C, ground (Paderno Meat Grinder, Padinox Inc., ON, Canada), aliquoted and freeze dried (FreeZone 12 L, Labconco Corp., MO, USA) for 48 h (−50°C and 0.12 mBar) followed by further homogenization (three 10 s pulses; Model 043-5781-8, Master Chef Elite Grinder, Trileaf Distribution, ON, Canada). The ventricle, liver, gut and white muscle samples were manually ground on liquid nitrogen with mortar and pestle. All samples were stored at −80°C until further analysis. Plasma was analyzed for triglyceride (TG) content using a commercial assay kit (10010303, Cayman Chemical, Ann Arbor, MI, USA).

*In vivo* protein synthesis rates in the ventricle, liver, gut and white muscle were estimated using the flooding dose technique (Cassidy et al., 2016; Lamarre et al., 2015). Briefly, 4 h prior to euthanization, fish were given a 1 ml per 100 g body mass intraperitoneal injection of 150 mmol l^−1^ of phenylalanine solution, which contained 50% deuterium labeled phenylalanine ([D5]-phenylalanine, 98%, Cambridge Isotope Laboratories, Inc., Tewksbury, MA, USA). Prior to sampling tissues, the abdominal cavity of the fish was rinsed with 0.8% saline solution to wash off any unabsorbed tracer. The fractional rate of protein synthesis (*K*_s_; % day^−1^) was then assessed as described in Lamarre et al. (2015).

Bomb calorimetry (6765 Combination Calorimeter, Parr Instrument Company, Moline, IL, USA) was used to determine the carcass energy density (kJ g^−1^ dry mass) as per the manufacturer's protocols (Brett et al., 1969).

A commercial assay kit (ab65336, Abcam Inc., ON, Canada) was used to quantify the TG contents of the liver, gut and white muscle.

The Bradford assay was used to quantify protein content in the liver, gut, white muscle and carcass. Briefly, dry (carcass; 0.0125±0.0007 g) or wet (liver, gut and white muscle; 0.05±0.005 g) tissue was added to 500 µl of homogenization buffer (50 mmol l^−1^ TRIS, 1 mmol l^−1^ EDTA, 3.5% NaCl, pH=7.9±0.05) and homogenized with a motorized micro pestle (2×30 s pulses; Cole-Parmer, Argos Technologies, QC, Canada) followed by sonication (2×10 s pulses; Q55 Sonicator, QSonica, Newtown, CT, USA). Then, 750 µl of homogenization buffer was added to each 1.5 ml tube and samples were vortexed and then centrifuged (10 min at 5000 ***g*** and 4°C; 5424R, Eppendorf AG, Germany). The supernatant was then diluted further (1:4 for gut, 1:8 for carcass, white muscle and liver), vortexed and assessed for protein content using Bradford reagent (B6919, Sigma, Sigma-Aldrich, ON, Canada) on a microplate reader at 595 nm (Epoch2 Microplate Reader, BioTek Instruments, Winooski, VT, USA).

### Data analysis

#### Spontaneous activity analysis

The daily spontaneous activity of brook char in experiment 1 was measured over a ∼24 h video recording at each temperature or day at 2°C using automated tracking software (ToxTrac, v2.84; Rodriguez et al., 2018). The first 2 h (07:00–09:00 h) of the recordings on each new day were removed from analysis to reduce the effect of disturbance from feeding that occurred within this time. ImageJ (v1.52a, National Institutes of Health, Bethesda, MD, USA; Schneider et al., 2012) was used to obtain the pixel-to-distance calibration required to make accurate distance moved calculations. Spontaneous activity in experiment 1 is expressed as the average speed over 24 h at each temperature or each day at 2°C. Average speed was calculated as the total distance moved by the fish in the recorded period divided by the corresponding duration and standardized to the length of each fish (average body lengths moved per minute, BL min^−1^).

The spontaneous activity of brook char in respirometers in experiment 2 was measured during the second ∼24 h of each 48-h respirometry measurement period (i.e. during the 24 h when *Ṁ*_O_2_standard_ and *Ṁ*_O_2_average_ were estimated) using automated tracking software (Ethovision XT v.16, Noldus Information Technology BV, The Netherlands). Activity level was quantified as the percentage of pixel change over 10 samples per second of video and averaged to one activity measurement for every *Ṁ*_O_2__ measurement. The percentage of pixel change was chosen as the unit of activity measurement because fish were confined in respirometers, so they occasionally had bouts of intense activity that did not result in a large distance travelled. Owing to an instance of a brief camera and infrared lighting failure while recording during the 60-day measurement, a total of 120 min of video was removed from the analysis. We also removed outright the activity measurements of eight individuals for one measurement day owing to excessive surface water movement from a misplaced air stone. Thus, the resulting sample sizes for fed at 8°C, starved at 8°C, fed at 2°C and starved at 2°C were 10, 12, 9 and 11 at time 0; 14, 14, 9 and 11 at time 1 (30 days); and 14, 14, 10 and 11 at time 2 (60 days) and time 3 (90 days), respectively.

#### Calculating lean body mass, Fulton's condition factor and *Q*_10_ values

Lean body mass (*M*_lean_) was calculated after 90 days using the body fat measurement (as a proportion; *F*_body_) and the following equation:
(1)

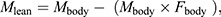
where *M*_body_ is body mass.

Fulton's condition factor (*K*) was calculated using the following equation:
(2)

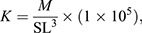
where *M* is mass (g) and SL is standard length (mm).

The *Q*_10_ values characterizing the thermal sensitivity of various rates were calculated using the following equation:
(3)

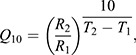
where *R*_1_ is the rate for a biological process at the lower temperature, *T*_1_, and *R*_2_ is the rate at the higher temperature, *T*_2_.

#### Calculating somatic indices

One-way linear models or generalized linear models (LMs or GLMs; see below) were constructed for each organ mass or length and were used to calculate a size-corrected organ mass for all treatment groups. The model residuals from the body mass or length term for each individual were added to the model estimated value at the average mass (0.152 kg) or length (225 mm; intestine length only) to obtain a size-adjusted organ mass or length for each individual. The adjusted organ masses were then divided by the average body mass or length and multiplied by 100 to generate the somatic index as a percentage. Adjusting organ mass to a common body mass in this way compensates for any deviation from a 1:1 mass or length scaling relationship, which is an assumption made when calculating somatic indices. Additionally, to examine how the heart, the central circulatory organ, and digestive organs changed relative to one another among treatments, we also calculated the gut:heart ratio by dividing total gut mass by ventricular mass. The gut:heart ratio can be useful to explore relative changes in organ sizes because it should be more independent of changes in body composition (e.g. fat accumulation or increased water content) than traditional somatic indices.

### Statistics

Statistical analysis was done using R (https://www.r-project.org/) in R Studio or Prism v.9 (GraphPad Software, San Diego, CA, USA), with data visualization completed using Prism v.9 (GraphPad Software). Statistical significance was accepted at *P*<0.05. All values are presented as means±s.e.m., unless otherwise noted.

#### Experiment 1: statistics for behavioural responses to cooling

Generalized linear mixed-effects models (GLMMs) were used to evaluate the effect of cooling on the spontaneous activity (family=Gamma, link=log) and food consumption (package ‘glmmTMB’: https://CRAN.R-project.org/package=glmmTMB; family=beta, link=logit) of brook char. The beta distribution does not recognize values of 0 or 1, so food consumption data were transformed using the following equation: *y*′=[*y*(*n*–1)+0.5]/*n*, where *y* is food consumption as a proportion and *n* is the sample size (Smithson and Verkuilen, 2006). The data were split into acute cooling (14°C to 2°C) and acclimating (2 weeks at 2°C) phases. During acute cooling, spontaneous activity and food consumption were modelled as a function of temperature with fish ID included as a random factor to account for repeated measures. During the 2-week acclimation at 2°C, spontaneous activity and food consumption were modelled as a function of acclimation duration with fish ID as a random factor. Type II Wald chi-square tests were then performed on all models using the ‘car’ package (https://CRAN.R-project.org/package=car) and *post hoc* multiple comparisons were performed using the ‘emmeans’ package (https://CRAN.R-project.org/package=emmeans) with Holm adjustments.

#### Experiment 2: statistics for metabolic performance and energetics

Data for experiment 2 were analyzed using LMs or linear mixed-effects models (LMMs) if there were repeated measures, unless parametric assumptions were not met, in which case the corresponding generalized model (GLM or GLMM; family=Gamma, link=log) was used. Each metric was modelled as a function of temperature, feeding status and time (if applicable), with lean body mass (90 day measurements only), body mass or body length as a covariate when applicable. Fish ID was included as a random factor for models with repeated measures (see Supplementary Materials and Methods for additional details and sample sizes). In each case, type II Wald chi-square tests were performed on all models using the ‘car’ package, followed by supervised *post hoc* comparisons of estimated marginal means (EMMs) with a Holm *post hoc* adjustment to account for multiple comparisons using the ‘emmeans’ package when significant effects or interactions were found.

The allometric scaling of *Ṁ*_O_2__ with body mass was accounted for in data presentation by adjusting *Ṁ*_O_2_standard_ and *Ṁ*_O_2_average_ to the average body mass. To do this, EMMs at the average mass were presented in place of means, or the residuals of the mass versus *Ṁ*_O_2__ relationship in each model were added to the predicted *Ṁ*_O_2_ _value from the model at the average mass (0.146 kg) to generated individual adjusted values for boxplots. Mass-specific values were then generated by dividing these adjusted *Ṁ*_O_2_ _values by the average mass (Gilbert, 2020). For the 90-day metabolic performance metrics, *Ṁ*_O_2__ was adjusted to the average lean body mass (0.154 kg) of the fish. This lean-mass adjustment was only done at 90 days, because it was the only time point at which we measured body fat.

## RESULTS

### Experiment 1: characterizing the effect of cooling on spontaneous activity and daily food consumption of brook char

**Fig. 1.**
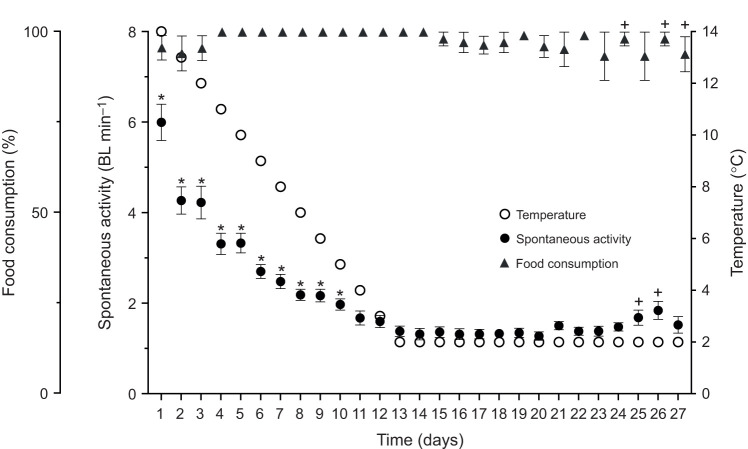
**Food consumption and spontaneous activity of juvenile brook char (experiment 1).** (A) Food consumption (closed triangles) and (B) spontaneous activity (closed circles) of fish were measured during acute cooling (1°C day^−1^; open circles) and subsequent holding at 2°C for 2 weeks. Food consumption is the percentage of the daily ration (0.5% body mass). Data are means±s.e.m. (*n*=15). Asterisks (*) and crosses (+) indicate significant differences relative to the first day at 2°C (day 13) for the period before or after that day, respectively (GLMMs and Type II Wald chi-square tests).

Daily food consumption remained high (>90%) during cooling (14°C to 2°C) (χ^2^_12,15_=7.55, *P*>0.05) and only minor day-to-day variation occurred over the 2 weeks at 2°C (χ^2^_14,15_=58.55, *P*<0.0001; Fig. 1). Spontaneous activity decreased by 77% (*Q*_10,14°C−2°C_=3.4; Table 2) with cooling from 14°C to 2°C (χ^2^_12,15_=859.43, *P*<0.0001; Fig. 1). Spontaneous activity was then maintained or slightly increased during the 2 weeks at 2°C (+10% from day 1 to day 10; χ^2^_14,15_=42.78, *P*<0.0001; Fig. 1).

**Table 2.**
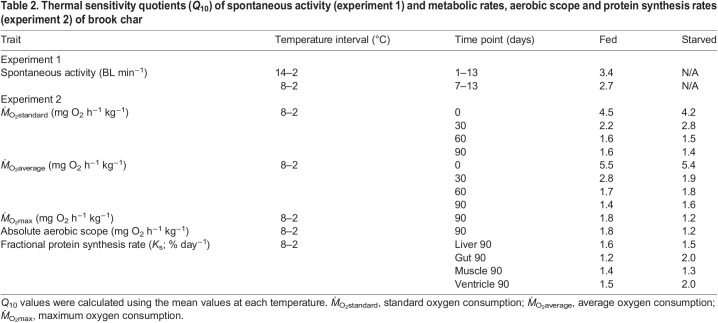
Thermal sensitivity quotients (*Q*_10_) of spontaneous activity (experiment 1) and metabolic rates, aerobic scope and protein synthesis rates (experiment 2) of brook char

### Experiment 2: the effects of prolonged starvation at 8°C or 2°C on brook char energetics and performance

#### Growth metrics

Body length, body mass and Fulton's condition factor (*K*) increased over the 90 day acclimation period in fed fish similarly at both temperatures, but length remained unchanged and mass and *K* decreased in starved fish (Table 1; Table S2).

#### Aerobic metabolism

**Fig. 2.**
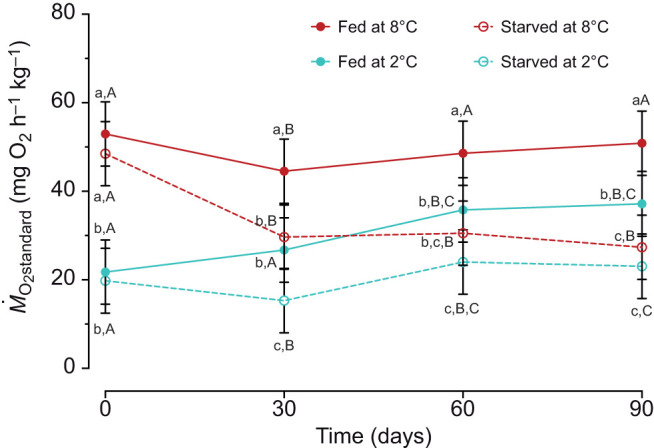
**The standard rate of oxygen consumption (*Ṁ*_O_2_standard_) of adult brook char over 90 days of acclimation (experiment 2).** Fish were acclimated to 2°C (teal) or held at 8°C (red; control) while fed daily (closed circles with solid line) or starved (open circles with dashed line). Treatments started on day 0; for cold acclimation, fish were acutely cooled (over ∼10 h) from 8°C to 2°C. Data are estimated marginal means (±s.e.m., *n*=10–14; see Materials and Methods for exact sample sizes) generated from a GLMM and adjusted to an average mass of 0.146 kg. Different lowercase letters represent significant differences between treatments within a time point, and different uppercase letters represent differences between time points within a treatment group (GLMMs and Type II Wald chi-square tests).

**Fig. 3.**
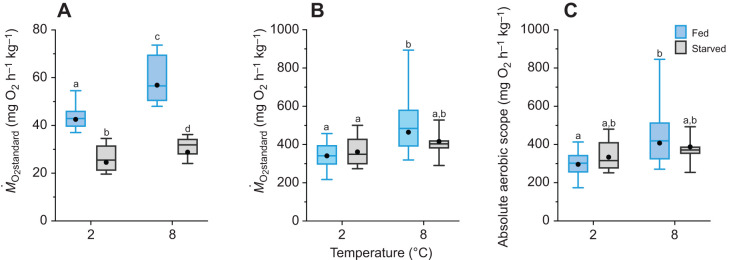
**Aerobic metabolic performance of adult brook char following 90 days at 2°C or 8°C while either fed or starved.** (A) Standard oxygen consumption (*Ṁ*_O_2_standard_), (B) maximum oxygen consumption (*Ṁ*_O_2_max_) and (C) absolute aerobic scope. Data are adjusted to an average lean body mass of 0.154 kg (see Materials and Methods). The boxplots represent the median and interquartile range, with whiskers indicating the 5–95% confidence interval; the filled black circle represents the estimated marginal mean (*n*=10–14; see Materials and Methods for exact sample sizes) from the corresponding GLM. Different letters indicate significant differences between treatment groups (GLMs and Type II Wald chi-square tests).

*Ṁ*_O_2_standard_ markedly decreased following initial acute cooling from 8°C to 2°C in both feeding treatments (Holm *post hoc*, *P*<0.0001; Fig. 2; Table S2), resulting in high *Q*_10_ values (4.2–4.5; Table 2). The effect of temperature and feeding treatment on *Ṁ*_O_2_standard_ varied over the experiment, resulting in significant two-way interactions of time with temperature and feeding treatment (Wald chi-square test, *P*<0.01 for both; Table S2). Specifically, *Ṁ*_O_2_standard_ at 8°C remained constant in fed fish over time, but decreased by 44% in starved fish (Fig. 2). At 2°C, *Ṁ*_O_2_standard_ in fed fish increased by 71% with acclimation (Fig. 2), but only by 17% in starved fish, with this increase occurring between 30 and 90 days (Holm *post hoc*, *P*<0.0001) (Fig. 2). Consequently, *Ṁ*_O_2_standard_ of starved fish at both temperatures was significantly lower than in fed fish after 90 days (–48% at 8°C and −40% at 2°C) (Figs 2 and 3A). The difference in *Ṁ*_O_2_standard_ between 8°C and 2°C decreased over time in both fed and starved fish (*Q*_10_=1.4–2.8; Table 2, Figs 2 and 3A); after 90 days *Ṁ*_O_2_standard_ for fed fish was significantly lower at 2°C than 8°C (Holm *post hoc*, *P*<0.001), but not for starved fish (Holm *post hoc*, *P*>0.05). *Ṁ*_O_2_average_ showed a similar pattern to *Ṁ*_O_2_standard_, with significant interactions between temperature and time as well as feeding status and time (Wald chi-square test, *P*<0.01 for both; Fig. S2A, Table S2). A minor exception is that at 90 days, *Ṁ*_O_2_average_ was similar in fish fed at 2°C and starved at 8°C (Holm *post hoc*, *P*>0.05).

*Ṁ*_O_2_max_ was higher in fed fish at 8°C than in fed or starved fish at 2°C (Holm *post hoc*, *P*<0.05; Table 2, Fig. 3B, Table S4). *Ṁ*_O_2_max_ was similar among starved fish at the two temperatures and fed fish at 2°C (Holm *post hoc*, *P*>0.05; Fig. 3B). AAS exhibited the same pattern, except the AAS of fed fish at 8°C was only significantly higher than fed fish at 2°C (Holm *post hoc*, *P*<0.01; Table 2, Fig. 3C; Table S4).

#### Median activity

Median activity of fish in the respirometers was very close to zero in all treatments at each time point, supporting the use of the 20th percentile approach to estimate *Ṁ*_O_2_standard_ as the fish were largely inactive (Fig. S2C).

#### *In vivo* protein synthesis rates

**Fig. 4.**
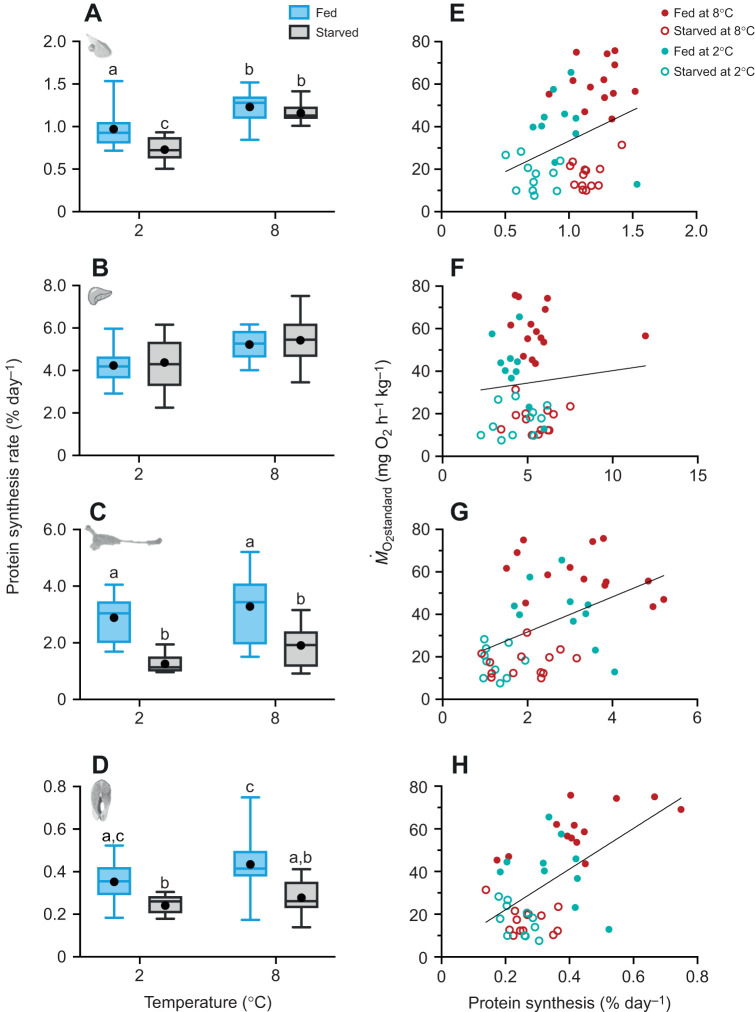
**Fractional rates of *in vivo* protein synthesis in brook char.** Rates are shown for (A) ventricle, (B) liver, (C) gut and (D) white muscle, along with their respective linear regression with *Ṁ*_O_2_standard_ (E–H) following 90 days at 2°C (teal) or 8°C (red) while either fed daily (blue; closed circles) or starved (grey; open circles). Boxplots represent the median and interquartile range, with whiskers indicating the 5–95% confidence interval; the filled black circle represents the mean (*n*=10–14; see Materials and Methods for exact sample sizes). Different letters represent significant differences between treatment groups (GLM or LM and Type II Wald chi-square tests). Linear regressions are shown for the ventricle (E; *P*<0.05, *R*^2^=0.116), liver (F; *P*>0.05, *R*^2^=0.007), gut (G; *P*<0.01, *R*^2^=0.205) and white muscle (H; *P*<0.0001, *R*^2^=0.329).

The *K*_s_ varied among treatment groups in an organ-specific manner, but decreased with temperature in all cases (*Q*_10_ range: 1.2–2.2; Wald chi-square test, *P*<0.05 for all; Table 2; Tables S5 and S6). Ventricle *K*_s_ decreased with starvation; however, this difference was only significant at 2°C (–25% at 2°C; Wald chi-square test, *P*=0.004; Fig. 4A; Table S4). Liver *K*_s_ was unaffected by feeding status (Wald chi-square test, *P*>0.05; Fig. 4B, Table S3). In contrast, gut *K*_s_ was markedly lower in starved fish (–42% at 8°C and −56% at 2°C; Holm *post hoc*, *P*<0.05; Fig. 4C; Table S4) as was muscle *K*_s_ (–36% at 8°C and −32% at 2°C; Fig. 4D, Table S3). *K*_s_ was positively correlated with *Ṁ*_O_2_standard_ in the white muscle, gut and ventricle (Fig. 4). White muscle had the strongest correlation to *Ṁ*_O_2_standard_ (*R*^2^=0.329, *P*<0.0001; Fig. 4H), followed by the gut (*R*^2^=0.205, *P*<0.01; Fig. 4G) and then the ventricle (*R*^2^=0.116, *P*<0.05; Fig. 4E), whereas the correlation was not significant for liver *K*_s_ (*R*^2^=0.007, *P*>0.05; Fig. 4F).

#### Carcass composition

Moisture content (% body mass) was, on average, 5% higher in starved fish than in fed fish (Holm *post hoc*, *P*<0.0001; Tables S1 and S4). Body fat was 25-27% lower in starved fish than in fed fish (Holm *post hoc*, *P*<0.01; Tables S1 and S4). Total relative carcass protein did not differ between treatment groups (Holm *post hoc*, *P*>0.05; Tables S1 and S3). Energy density was 9–13% lower in starved fish than in fed fish (Holm *post hoc*, *P*<0.01; Tables S1 and S4).

#### Organ sizes and somatic indices

**Fig. 5.**
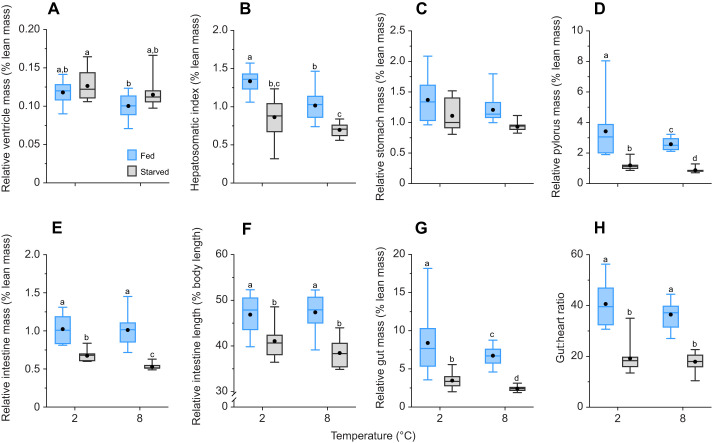
**Brook char organ-somatic indices following 90 days at 2°C or 8°C while either fed daily or starved.** Data for the (A) ventricle, (B) liver, (C) stomach, (D) pylorus, (E,F) intestine, (G) total gut and (H) gut:heart ratio are shown as boxplots representing the median and interquartile range, with whiskers indicating the 5–95% confidence interval; the filled black circle represents the mean (*n*=10–15; see Materials and Methods for exact sample sizes). Different letters represent significant differences between treatment groups within a panel (GLM or LM and Type II Wald chi-square tests).

After accounting for differences in body size, the mass of the ventricle was only different between fed fish at 8°C and starved at 2°C (Holm *post hoc*, *P*<0.01; Fig. 5A; Table S4). Liver mass was significantly smaller in starved fish compared with fed fish (Holm *post hoc*, *P*<0.01; Fig. 5B; Table S4). The mass of the stomach did not differ among treatment groups (Holm *post hoc*, *P*>0.05; Fig. 5C; Table S3). Pylorus masses and total gut masses were significantly different between all treatment groups, with fed fish at 2°C having the greatest masses (Holm *post hoc*, *P*<0.05 and *P*<0.01, respectively), and starved fish at 2°C having the lowest masses (Holm *post hoc*, *P*<0.05 and *P*<0.01, respectively) (Fig. 5D,G; Table S3). Intestine mass was lower at 8°C than at 2°C, but only in starved fish, resulting in a significant interaction between temperature and feeding status (Table S3). Additionally, intestine mass was 34% (2°C) and 48% (8°C) smaller in starved fish than in fed fish (Wald chi-square test, *P*<0.001; Fig. 5E; Table S3). Intestine length and the gut:heart ratio were significantly lower in starved fish than in fed fish (Holm *post hoc*, *P*<0.01 and *P*<0.0001, respectively) (Fig. 5F,H; Table S4).

#### Organ triglyceride and protein content

**Fig. 6.**
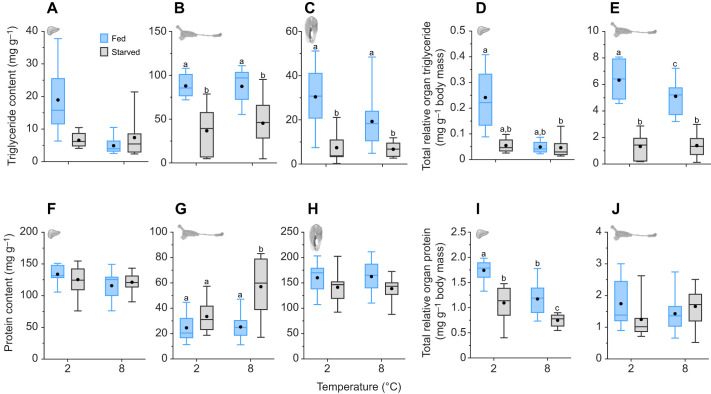
**Organ-specific triglyceride (TG) and protein content in brook char following 90 days of daily feeding or starvation at 2°C or 8°C.** The TG and protein contents are presented for each organ (A–C, F–H, respectively); also shown is the total relative organ TG and protein content (D–E, I–J, respectively). Total relative organ tissue TG and protein were calculated as the total TG or protein (TG or protein concentration multiplied by organ mass) divided by lean body mass. Data for the (A,D,F,I) liver, (B,E,G,J) gut and (C,H) white muscle are shown as boxplots representing the median and interquartile range, with whiskers indicating the 5–95% confidence interval; the filled black circle represents the mean (*n*=10–15; see Materials and Methods for exact sample sizes). Different letters represent significant differences between treatment groups (GLM or LM and Type II Wald chi-square tests).

The TG and protein contents varied among treatment groups in an organ-specific manner. Liver TG content was unaffected by temperature and feeding status (Wald chi-square test, *P*>0.05 for both; Fig. 6A; Table S3). Gut TG content was 58% (2°C) and 49% (8°C) lower in starved fish than in fed fish (Holm *post hoc*, *P*<0.0001; Fig. 6B; Table S4). White muscle TG content was also lower in starved fish, albeit to a lesser extent (32% at 2°C and 18% at 8°C; Holm *post hoc*, *P*<0.001; Fig. 6C; Table S3). Total relative liver TG (i.e. how much of the body mass is made up of liver TG) was significantly lower in starved fish at 8°C than in fed fish at 2°C (Holm *post hoc*, *P*<0.01; Fig. 6D; Table S3). Total relative gut TG was 73–79% lower in starved fish than in fed fish at both temperatures (Holm *post hoc*, *P*<0.0001) and higher at 8°C than at 2°C, but only in fed fish, resulting in a significant interaction between temperature and feeding treatment (Holm *post hoc*, *P*<0.05; Fig. 6E; Table S4).

Liver and white muscle protein contents did not differ between treatment groups (Wald chi-square test, *P*>0.05 and Holm *post hoc*, *P*>0.05, respectively; Fig. 6F,H; Table S4). However, total relative liver protein was significantly lower in starved fish at 8°C (Holm *post hoc*, *P*<0.01) and significantly higher in fed fish at 2°C (Holm *post hoc*, *P*<0.001) than all other treatment groups (Fig. 6I; Table S4). Gut protein content was significantly higher in fish starved at 8°C than all other groups (Home *post hoc*, *P*<0.001), though total relative gut protein did not differ between treatment groups (Holm *post hoc*, *P*>0.05) (Fig. 6G,J; Table S4).

#### Blood parameters

Plasma glucose was similar among treatment groups, except it was lower in starved fish at 8°C compared with those fed at 2°C (Holm *post hoc*, *P*<0.05; Tables S1 and S6). Haematocrit did not differ among treatment groups (Holm *post hoc*, *P*>0.05; Tables S1 and S6). Plasma TG content at 2°C was 56% lower in starved than in fed fish (Holm *post hoc*, *P*<0.05; Tables S1 and S6).

## DISCUSSION

When temperatures drop and food becomes scarce over winter, winter-active species (e.g. many salmonids) can face trade-offs between energy conservation and the maintenance of metabolic performance (Auer et al., 2020). Our findings support the hypothesis that winter-active fishes employ behavioural and physiological flexibility in response to cold and food scarcity that allows them to conserve energy while also protecting metabolic performance. Most notably, we found that brook char can adjust their metabolic floor (*Ṁ*_O_2_standard_) in a food- and temperature-dependent manner to balance the competing needs for energy conservation and thermal compensation. Essentially, brook char greatly depressed *Ṁ*_O_2_standard_ to conserve energy in the absence of food, underwent marked thermal compensation by increasing *Ṁ*_O_2_standard_ during cold acclimation, and did both simultaneously when these challenges occurred together, all with no apparent trade-off in *Ṁ*_O_2_max_ or AAS. Our assertion that selective metabolic depression and thermal compensation occur simultaneously is supported by two key findings. First, the reduction in *Ṁ*_O_2_standard_ observed during food deprivation at 8°C was not apparent at 2°C, even though both groups had similar tissue-level starvation responses (reductions in *K*_s_ and the atrophy of non-essential tissues), which should depress *Ṁ*_O_2_standard_. Second, the final thermal sensitivity quotients between starved fish at 8°C and 2°C were low and similar to those of fed fish, suggesting that significant thermal compensation had occurred in brook char in the absence of food despite the final *Ṁ*_O_2_standard_ still being much lower in starved than in fed fish at 2°C. Our findings from the biochemical to whole-animal level indicate that brook char are well suited to cope with variation in winter conditions, show that the apparent extent of metabolic thermal compensation can be food dependent, and provide additional evidence for common metabolic overwintering mechanisms among vertebrates adapted to extreme seasonality.

### Activity, feeding and growth in the cold

Brook char, like other salmonids, often remain active and feeding throughout the winter in the wild (Cunjak and Power, 1987; Cunjak et al., 1987; Fraser et al., 1993) and in the laboratory (Helland et al., 2011). Similarly, we observed continued feeding, growth and increased body condition even during prolonged holding at 2°C. Activity did decline with cooling at a rate suggesting a primarily passive physicochemical effect (*Q*_10,14°C–2°C_=3.4) (Reeve et al., 2022). Despite this decline, brook char activity at 2°C remained elevated compared with winter-dormant or -lethargic species (Reeve et al., 2022), in which active behavioural responses such as sheltering contribute to more pronounced inactivity. Nonetheless, the observed activity reductions in brook char would greatly reduce routine energy demands, supporting the notion that activity decrement is a common energy conservation mechanism among overwintering fishes (Reeve et al., 2022).

### Plasticity of resting metabolic demands

When ectotherms are cooled, the passive slowing of resting metabolic demands via Arrhenius effects is generally associated with *Q*_10_=2–3 (Clarke and Johnston, 1999). Following a rapid cooling (6°C over ∼10 h) to 2°C, brook char *Ṁ*_O_2_standard_ markedly declined, with high *Q*_10_ values (4.2–4.5). A high *Q*_10_ (e.g. >3.5) for *Ṁ*_O_2_standard_ during cooling can indicate an active metabolic rate depression (Geiser, 2016; Reeve et al., 2022; Staples, 2016). However, the high thermal sensitivity seen here is more parsimonious with an acute metabolic constraint, as *Ṁ*_O_2_standard_ greatly increased during subsequent cold acclimation, resulting in typical Arrhenius *Q*_10_ values of 2–3 by 30 days and ∼1.6 by 60 days. Thus, we found strong metabolic thermal compensation, not depression. The thermal compensation of *Ṁ*_O_2_standard_ in starved fish appeared less pronounced, likely because thermal compensation was occurring alongside processes that reduce *Ṁ*_O_2_standard_ during food deprivation. Thermal compensation during cold acclimation has been demonstrated at sub-organismal levels in many fishes (Dean, 1969; Guderley, 1990; Hochachka and Hayes, 1962; Orczewska et al., 2010), although more rarely at the whole-animal level (Peterson and Anderson, 1969). For example, oxygen uptake of brook char muscle homogenates over doubled following cold acclimation (4°C for at least 2 weeks), though *Q*_10_ values remained relatively high (>3) (Hochachka and Hayes, 1962). At the whole-animal level, the *Q*_10_ for *Ṁ*_O_2_standard_ from 18°C to 6°C fell from 3.2 in Atlantic salmon acclimated to 18°C to 2.4 following 1 month of acclimation to 6°C (Peterson and Anderson, 1969). We demonstrated a much more pronounced compensation, with post-acclimation *Q*_10_ values (∼1.5) that are among the lowest for resting metabolism in teleosts (e.g. rainbow trout, *Oncorhynchus mykiss*: *Q*_10,5°C–26°C_=2.02; lake char: *Q*_10,9.1°C–22.1°C_=2.74; Bokma, 2004; Watanabe and Payne, 2023; White et al., 2006). This remarkable compensation during cold exposure has not been previously documented in brook char, likely because studies rarely include acute and acclimated exposures and may not use sufficient acclimation durations (Havird et al., 2020). Indeed, our results demonstrate that common acclimation times of 3 to 4 weeks do not capture the full extent of cold acclimation in brook char. The pronounced cold acclimation responses we found here may be more common among winter-active species (e.g. acclimated lumpfish, *Cyclopterus lumpus*: *Q*_10,3°C–15°C_=1.7; or Arctic grayling, *Thymallus arcticus*: *Q*_10,4°C–12°C_=1.9; Bokma, 2004) than current literature suggests (Abe et al., 2019).

In contrast to thermal compensation in the cold, resting metabolic demands are commonly reduced in animals faced with food deprivation, including in fishes (Auer et al., 2016b; Hvas et al., 2020; O'Connor et al., 2000; Van Leeuwen et al., 2012). For example, following 10 days of starvation at 14°C, juvenile coho salmon (*Oncorhynchus kisutch*) downregulated their *Ṁ*_O_2_standard_ by 26–29% (Van Leeuwen et al., 2012). Here, brook char showed a similar initial reduction and by 90 days of starvation, *Ṁ*_O_2_standard_ was >40% lower in starved fish. This reduction was accompanied by a modest loss of body mass, but substantial reductions in body fat and energy density, alongside an increase in moisture content, which are all typical vertebrate responses to starvation (Brett et al., 1969; Cassidy et al., 2016; Jobling et al., 1998a; Navarro and Gutierrez, 1995; Secor and Carey, 2016). This pattern was primarily driven by a depletion of lipid stores, specifically TG in the white muscle and gut, with little to no reduction in protein content. Thus, even after 90 days of food absence, brook char likely remained in the second phase of starvation (lipid use and protein sparing) (Bar, 2014; Bar and Volkoff, 2012; Navarro and Gutierrez, 1995; Secor and Carey, 2016), as seen in Atlantic salmon (Foda, 1975). Although TG levels were approaching zero in some individuals, the lack of significant change in protein content suggests that brook char could have survived longer without food, albeit in a critical phase of starvation (phase three: protein catabolism). Thus, the observed depression of *Ṁ*_O_2_standard_ during starvation and corresponding energy savings (Van Leeuwen et al., 2012) was likely essential to the starvation tolerance seen here.

### Energy conservation through tissue selective atrophy and depression of protein synthesis

Animals that experience energy deficits commonly reduce their resting metabolic rate by downregulating energy demands in non-critical energy sinks such as the maintenance of non-essential tissues or protein synthesis (Cassidy et al., 2016; Dabrowski and Guderley, 2003; Fedorov et al., 2009; Méndez and Wieser, 1993; Tøien et al., 2011). Gut atrophy (i.e. changes in mass or length), in particular, is a common adaptive energy conservation response to starvation, given the gut's high metabolism and its disuse when not feeding. Gut mass in starved brook char decreased substantially at both 2°C and 8°C, similar to other *Salvelinus* spp. [e.g. Arctic char and Dolly Varden (*Salvelinus malma*)] in response to starvation (Armstrong and Bond, 2013; Jobling et al., 1998b; Jørgensen et al., 1997). Interestingly, unlike in Atlantic salmon and Dolly Varden, which endure long periods of starvation associated with anadromy (Armstrong and Bond, 2013; Martin et al., 1993), we did not observe a decrease in brook char stomach mass. Brook char will feed opportunistically in winter (Cunjak and Power, 1987), so maintaining the stomach may be worth the additional cost (Cunjak et al., 1987). The gut atrophy observed in starved brook char corresponded with a large reduction in gut *K*_s_, similar to that seen in rainbow trout following only 6 days of food deprivation (McMillan and Houlihan, 1989). The substantial combined atrophy and reduction of *K*_s_ indicate that the gut is a major site of energy savings during starvation. Although this downregulation obviously limits digestive capacity, these systems may be able to recover quickly, given that resting metabolic rates of Atlantic salmon returned to control levels following just 1 week of feeding (Hvas et al., 2020) and that compensatory growth following food deprivation is common (Cassidy et al., 2018; Davis and Gaylord, 2011).

The liver is a major site of protein metabolism and a site of glycogen and lipid loss during energy restriction (Armstrong and Bond, 2013; Dabrowski and Guderley, 2003; Lahnsteiner, 2022). We found that livers were smaller in starved fish at both 2°C and 8°C, which has energetic implications when considering liver *K*_s_. We found no change in liver *K*_s_ following 90 days of starvation in brook char. However, livers were smaller in starved fish relative to their body mass with no change in protein content, so total relative liver *K*_s_ would have been lower in starved fish. The response of liver *K*_s_ to starvation in salmonids varies among studies, possibly as a result of differences in the ration size for fed fish (McMillan and Houlihan, 1992).

While white muscle *K*_s_ values are much lower than liver or gut, white muscle makes up ∼50–60% of the body mass. Therefore, its overall contribution to whole-animal energy expenditure is significant (Milligan, 1996). Studies in Arctic char and rainbow trout have reported reductions in white muscle *K*_s_ ranging from 72 to 76% with starvation (Cassidy et al., 2016; Loughna and Goldspink, 1984; McMillan and Houlihan, 1989; Smith, 1981). Here, brook char reduced white muscle *K*_s_ by 31–35%. In contrast, ventricular *K*_s_ did not change with starvation in Arctic char and rainbow trout (Cassidy et al., 2016; McMillan and Houlihan, 1989), and we saw minor (at 2°C) or no (8°C) significant change in brook char. The lack of, or relatively minor reduction in, ventricular *K*_s_ in our study agrees with the absence of ventricular atrophy and is consistent with the heart's essential function and relatively slow cell turnover rates (Bergmann et al., 2009; Secor and Carey, 2016).

We focused on organ-specific changes in *K*_s_ and mass because they influence whole-animal metabolism and growth (Armstrong and Bond, 2013; Cassidy et al., 2016; Houlihan et al., 1995; Jørgensen et al., 1997; Loughna and Goldspink, 1984; Zaldúa and Naya, 2014). Protein synthesis alone may account for 20–50% of resting metabolic demands in growing fish (Carter and Houlihan, 2001). Indeed, protein synthesis of the white muscle, and to a lesser extent of the gut and ventricle, were positively correlated with *Ṁ*_O_2_standard_ in brook char. Although protein degradation rates were not estimated here, they should be investigated further as the balance between synthesis and degradation would influence the extent of atrophy observed. Protein degradation rates are reported to increase in the intestine, liver and white muscle with starvation in fishes (Cassidy et al., 2016; Krogdahl and Bakke-McKellep, 2005; Navarro and Gutierrez, 1995; Smith, 1981).

Overall, our results demonstrate that brook char downregulate *K*_s_ in an organ-specific manner and possess energy-saving digestive flexibility in response to starvation at 2°C and 8°C. Organ-specific *K*_s_ did decrease with temperature, which would confer additional energy savings in the cold. However, *Q*_10_ values were low (1.2 to 2.2) within feeding treatments, suggesting a thermal compensation of *K*_s_ during cold acclimation independent of the starvation-induced reductions, consistent with our findings for *Ṁ*_O_2_standard_. In combination with the processes we identified as helping reduce *Ṁ*_O_2_standard_, other studies have shown that increased mitochondrial efficiency (i.e. less proton leak) or reductions in mitochondrial abundance (Guderley and St-Pierre, 2002; Salin et al., 2018; Secor and Carey, 2016; Thoral et al., 2023) can contribute greatly to reductions in *Ṁ*_O_2_standard_ when food is limited.

### Conservation of aerobic metabolic performance during starvation

Like other levels of metabolic performance, *Ṁ*_O_2_max_ often decreases with cooling (Durhack et al., 2021; Mackey et al., 2021), but the responses of *Ṁ*_O_2_max_ to nutritional status are less well established. If starvation constrained aerobic performance, it could limit capacity for critical tasks such as predator avoidance, migration, and food acquisition and growth when resources return (Auer et al., 2015). Such a constraint is conceptually possible given the starvation-induced decrease of *Ṁ*_O_2_standard_ and the traditional view that *Ṁ*_O_2_standard_ and *Ṁ*_O_2_max_ are coupled (Bennett and Ruben, 1979). Conservation of *Ṁ*_O_2_max_ and aerobic performance has been observed in multiple other salmonids with food deprivation ranging from 1 to 4 weeks (Alsop and Wood, 1997; Hvas, 2022; Thorarensen and Farrell, 2006), but these studies did not explicitly assess *Ṁ*_O_2_standard_ alongside maximal performance. Here, even following a much longer 90-day food deprivation, we explicitly demonstrated that brook char maintained *Ṁ*_O_2_max_, and thus AAS, during starvation while simultaneously reducing *Ṁ*_O_2_standard_. This conservation of aerobic capacity during starvation was associated with the maintenance of plasma glucose, blood–oxygen carrying capacity (inferred from haematocrit) and ventricle mass, with minor or no reduction in ventricular *K*_s_, all of which likely contribute to the maintenance of peak aerobic performance (Gallaugher et al., 1995, 2001; Secor and Carey, 2016). Thus, we show that the responses of *Ṁ*_O_2_standard_ and *Ṁ*_O_2_max_ to food availability at winter-relevant temperatures in brook char can become decoupled to maintain aerobic scope. This uncoupling is likely possible because energy savings occurred in processes unrelated to peak exercise induced aerobic performance (e.g. gut size, gut protein synthesis). Our findings in brook char under winter-relevant stressors represent a new environmental context consistent with a growing understanding that maintenance of maximum aerobic performance can be decoupled from resting metabolic rate to varying extents among vertebrates, including other fishes (Armstrong and Bond, 2013; Auer et al., 2016a; Cassidy et al., 2016; Hvas, 2022), amphibians (Naya et al., 2009), reptiles (Secor, 2008), birds (Barceló et al., 2017; Funes et al., 2014) and mammals (Fedorov et al., 2009; Karasov and Douglas, 2013; Lignot, 2012; Secor and Carey, 2016; Tøien et al., 2011). Nonetheless, other limitations imposed by food deprivation and cold on metabolic fuel availability (e.g. reductions of glycogen and lipid reserves) and changes in muscle function could compromise endurance (hours to days), swimming performance and other energetically expensive processes (e.g. reproduction).

### Conclusions

Using an integrative examination of energetic responses to prolonged simulated winter conditions, we provide evidence that winter-active fishes, such as brook char, balance the maintenance of a high-capacity phenotype with energy conservation to survive winter and presumably improve subsequent individual fitness. This remarkable plasticity was observed despite fish being from a captive breeding population, and so responses in strains or species (e.g. Arctic char) that more routinely encounter food restriction may be even more pronounced (Turko et al., 2023). Understanding the overwintering physiology of winter-active species is important as ongoing changes in winter conditions, including warming and phenological shifts, could exacerbate energy depletion (Morash et al., 2021), altering the efficacy of current energy conservation strategies. Brook char showed an impressive ability to assimilate food and compensate for prolonged exposure to frigid temperature, while also displaying strong starvation tolerance (even at a warmer temperature) via an active depression of *Ṁ*_O_2_standard_. This great bioenergetic flexibility to exploit or tolerate shifting winter food availability and temperatures exemplifies the robust plasticity of *Salvelinus* fishes that helps explain their success across diverse habitats and may indicate their potential resilience to future winter conditions (Armstrong and Bond, 2013; Dutil, 1986; Gilbert and Farrell, 2021; Hutchings, 1996; MacCrimmon and Campbell, 1969; Muir et al., 2016).

The energetic flexibility of brook char in response to cold and starvation also holds important implications for bioenergetic modelling. Bioenergetics models are commonly used to estimate energy needs and growth of individuals and populations under varying environmental conditions, informing management and conservation (Brownscombe et al., 2022; Little et al., 2020). However, bioenergetics models currently do not account for potential reductions in resting metabolic demands during periods of restricted feeding. Our findings suggest that the inclusion of variation in metabolism associated with food restriction could markedly improve bioenergetic models.

The present findings also highlight the need for studies, like ours, that combine multiple factors over more ecologically relevant time courses to properly understand both acute and prolonged responses to environmental change (Havird et al., 2020; Huey and Buckley, 2022). Such experimental designs better capture the temporal dynamics of acclimation, which is particularly important when considering seasonal change that can occur over months rather than weeks. For example, the *Ṁ*_O_2_standard_ of brook char had only reached a steady state of cold acclimation by 60 days, well beyond typically used acclimation durations (∼4 weeks; 30 days). Additionally, multi-factorial designs can reveal unexpected phenotypic outcomes of changing environments, such as an apparent balancing of thermal compensation and starvation-induced reductions in *Ṁ*_O_2_standard_ of brook char. We recommend that studies on thermal plasticity consider the context of co-varying environmental factors and use experimental time scales most relevant to the animal in the wild.

## Supplementary Material

10.1242/jexbio.246743_sup1 Supplementary information
