# Supplementary material for: Environmental variation associated with overwintering elicits marked metabolic plasticity in a temperate salmonid, Salvelinus fontinalis
Source: J Exp Biol. 2024 Feb 12;227(3):jeb246743. doi: 10.1242/jeb.246743 (PMC10911287; doi:10.1242/jeb.246743)
Supplement: Supplementary information [file jexbio-227-246743-s1.pdf]

Journal of Experimental Biology • Supplementary information

Supplementary Materials and Methods

Calculating average and median activity

The average and median activity of each individual at each of the four time points was calculated by taking the mean or the median of all spontaneous activity measurements over a given 24-hour measurement period. Average activity was heavily skewed by brief bouts of high activity that had a transient effect on $\dot{M}O_2$. Therefore, median activity was also examined to better understand more typical activity levels. Median activity can indicate if the fish are mostly inactive while in the respirometers, which is helpful for ensuring that $\dot{M}O_{2standard}$ estimates are actually reflecting $\dot{M}O_2$ at zero activity. We did not explore or interpret the average activity values as a representation of routine voluntary activity like in Experiment 1 as fish in Experiment 2 were confined in the respirometers and were not fed during these measurements.

Validation of $\dot{M}O_{2standard}$ estimates

We measured spontaneous activity simultaneously with $\dot{M}O_2$, a method that allows us to account for any variation in activity between treatments (Reeve et al., 2022; Speers-Roesch et al., 2018). Fish at warmer temperatures and fish that are fed may inherently have higher activity levels which could result in an overestimate of $\dot{M}O_{2standard}$ using traditional methods, which could then lead to the identification of a greater difference in resting metabolic demands between treatment groups than what actually exists (Speers-Roesch et al., 2018). Thus, we validated the $\dot{M}O_{2standard}$ estimates obtained through a traditional lowest approach (20th percentile of all $\dot{M}O_2$ values) (Chabot et al., 2016b) using two other methods that involved estimating $\dot{M}O_2$ when activity was zero. First, we conducted linear regressions using Prism v.9 (GraphPad Statistical Software, San Diego, USA) for each treatment group with each individual's $\dot{M}O_2$ estimates as a

function of the corresponding spontaneous activity estimates over each 24-hour measurement period at each of the four time points (Figure S1). This regression provided a y-intercept ± S.E.M. for the relationship between $\dot{M}O_2$ and activity which was considered as the $\dot{M}O_2$ at zero activity for each treatment group (Figure S1). Second, we generated an estimated marginal means of log($\dot{M}O_2$) at zero activity using generalized linear mixed effects models (family=Gamma, link=log) in R Studio (Core Team, 2014) with $\dot{M}O_2$ (mgO_2 h^{-1}) as a function of spontaneous activity, temperature and treatment, with body mass included as a covariate and fish ID as a random factor. We took the antilog of the estimated marginal means of $\dot{M}O_{2standard}$ and its standard error to obtain an estimate of $\dot{M}O_{2standard}$ for comparison. We also compared these values to those calculated by the commonly used method of adjusting $\dot{M}O_{2standard}$ to each individual's body mass (or lean mass when available).

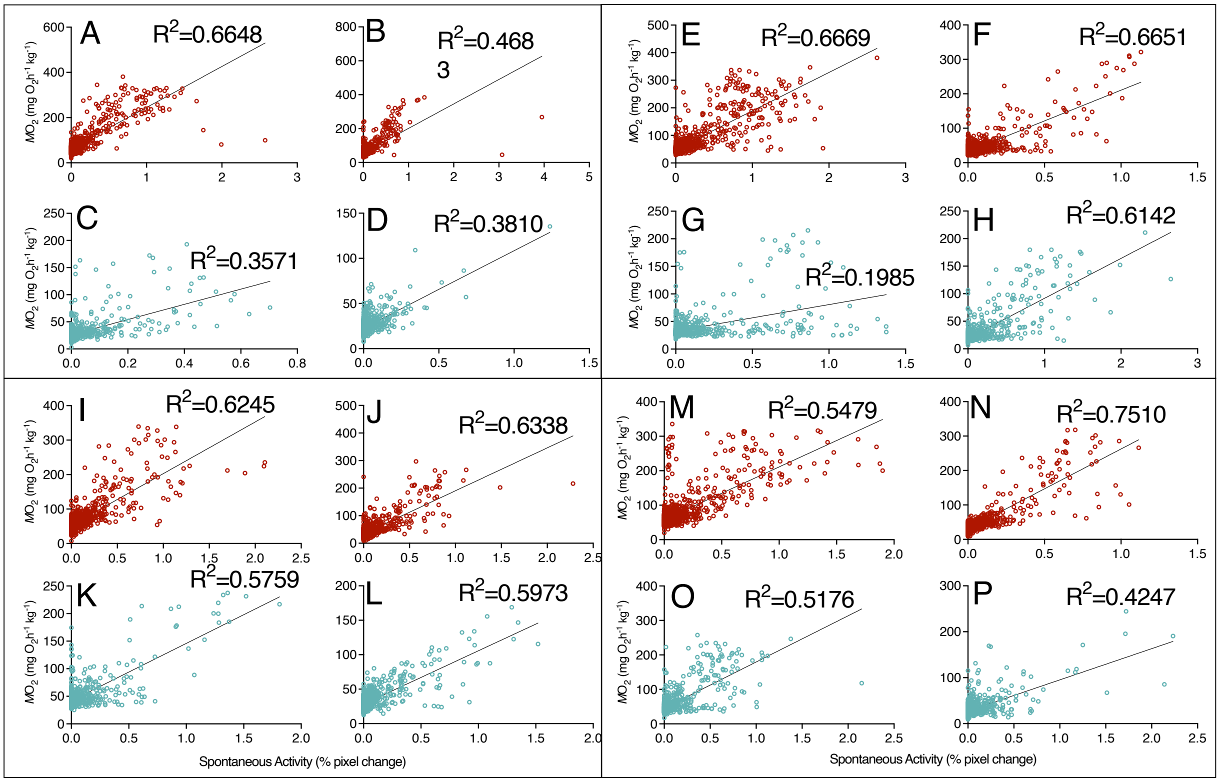

Fig. S1. Linear regressions between spontaneous activity and $\dot{M}O_2$ at the initial (day 0; top left quadrant), 30-day (top right quadrant), 60-day (bottom left quadrant) and 90-day (bottom right quadrant) measurements (Experiment 2). Data are shown for fed (A,C,E,G,I,K,M,O) and starved (B,D,F,H,J,L,N,P) fish at 2°C (blue circles; C,D,G,H,K,L,O,P) and 8°C (red circles; A,B,E,F,I,J,M,N). Linear regressions were performed using Prism v.9 (GraphPad Statistical Software, San Diego, USA) with the $\dot{M}O_2$ measurements of each individual related to the corresponding spontaneous activity estimate over a 24-hour measurement period to obtain a y-intercept which was used as an estimate of $\dot{M}O_{2standard}$ at 0 activity for each treatment group. Note that axes are on different scales because of the range in absolute values among treatments.

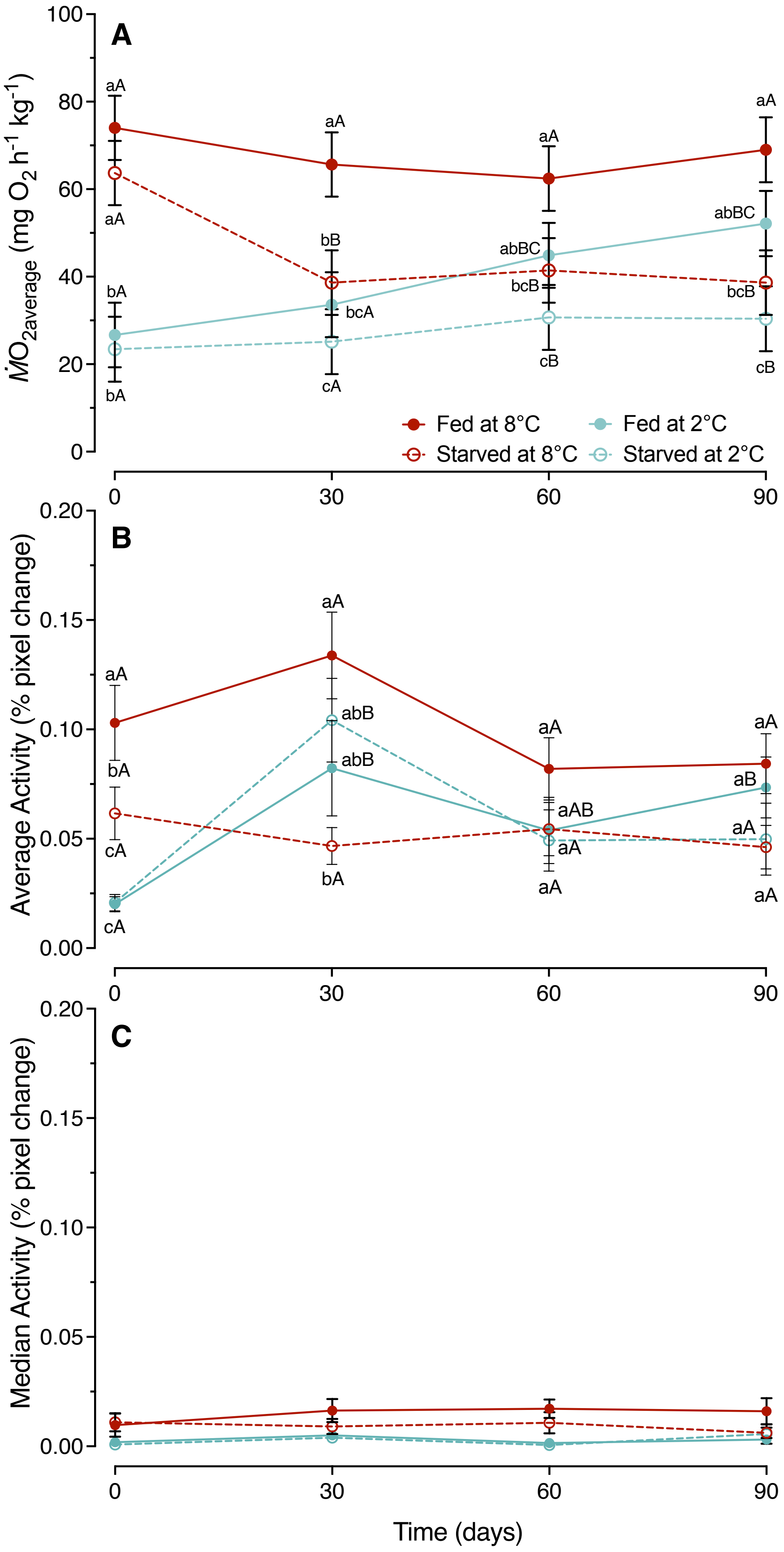
A
B
C
$\dot{M}O_{2average}$ (mg O_2 h^{-1} kg^{-1})
Average Activity (% pixel change)
Median Activity (% pixel change)
Time (days)
Fed at 8°C
Starved at 8°C
Fed at 2°C
Starved at 2°C
aA
bA
bB
bcA
cA
abBC
bcB
cB
abB
aB
aAB

Fig. S2. Average oxygen consumption ($\dot{M}O_{2average}$, A), average activity (B) and median activity (C) of adult brook char (Experiment 2) exposed to either 2°C (blue) or 8°C (red) while fed (closed circles and solid line) or starved (open circles and dotted line) over 90 days. Fish were either maintained at 8°C or acutely cooled from 8°C to 2°C (day 0) and subsequent cold acclimated for 90 days. Activity data was taken from a 24-hour measurement period, in which oxygen consumption ($\dot{M}O_2$) was measured simultaneously. Different lowercase letters represent significant differences between the treatment groups within each time point and different uppercase letters represent significant differences across time points within a treatment group (GLMM and Type II Wald chi-square test; Table S2). Median activity was lower at 2°C than at 8°C however, no specific pairwise comparisons were significant (LMM and Type II Wald chi-square test; Table S2).

Table S1. Blood parameters and carcass composition of adult brook char (Experiment 2) measured after 90 days of exposure to 2°C or 8°C while either fed (0.5% body mass ration) or starved. Glucose and triglyceride were measured in plasma.

	**Fed at 8°C n=14 (n=10 for [triglyceride])**	**Starved at 8°C n=15 (n=14 for hematocrit, n=10 for [triglyceride])**	**Fed at 2°C n=10**	**Starved at 2°C n=11 (n=10 for [triglyceride])**
**Glucose**	4.09 ± 0.11[ab]	3.63 ± 0.11[a]	4.28 ± 0.22[b]	3.79 ± 0.18[ab]
**Hematocrit (%)**	35.47 ± 0.84[a]	32.52 ± 0.92[a]	33.89 ± 1.65[a]	31.65 ± 1.01[a]
**Triglyceride (mg dL^{-1})**	234.79 ± 30.91[ab]	212.11 ± 23.28[ab]	269.64 ± 41.45[a]	119.79 ± 23.82[b]
**Moisture Content (%)**	72.38 ± 0.20[a]	76.30 ± 0.35[b]	72.46 ± 0.48[a]	76.68 ± 0.58[b]
**Body Fat (%)**	3.46 ± 0.08[a]	2.53 ± 0.10[b]	3.44 ± 0.15[a]	2.59 ± 0.19[b]
**Total Relative Carcass Protein (mg g body $mass^{-1}$)**	192.86 ± 23.34[a]	253.00 ± 30.48[a]	249.36 ± 45.18[a]	272.04 ± 40.50[a]
**Energy Density (kJ/g)**	24.83 ± 0.38[a]	22.65 ± 0.20[b]	26.10 ± 0.46[a]	22.68 ± 0.61[b]

Data are means ± S.E.M. Significant differences between treatment groups are indicated by different letters.

Journal of Experimental Biology • Supplementary information

Table S2. (See next page) Summary of statistical outputs obtained from generalized linear mixed effects models (GLMM; family=Gamma, link=log) or linear mixed effects models (LMM; median activity) examining the effects of temperature (2°C or 8°C), prolonged starvation, time under treatment, and their interactions on energetic and morphological variables of adult brook char (Experiment 2). These analyses correspond with the data shown in Table 1 (main text), Figure S2 and Figure 2 (main text). Significance ($p<0.05$) was assessed using type II Wald chi-square tests and is indicated in bold.

	**Temperature** (df=1)		**Feeding Status** (df=1)		**Time** (df=3)		**Mass** (df=1)		**Temperature: Treatment** (df=1)		**Temperature: Time** (df=3)		**Treatment: Time** (df=3)		**3-way Interaction** (df = 3)	
	X^2	p	X^2	p	X^2	p	X^2	p	X^2	p	X^2	p	X^2	p	X^2	p
**Length**	0.5	0.47	0.1	0.81	327. 6	**<0.01**			2.9	0.09	1.2	0.76	291.2	**<0.01**	2.9	0.42
**Body Mass**	0.4	0.54	1.4	0.24	545. 3	**<0.01**			1.6	0.21	4.9	0.18	2005. 5	**<0.01**	12.9	**0.01**
**Body Condition**	0.02	0.89	8.8	**<0.01**	44.6	**<0.01**			1.2	0.27	6.7	0.08	229.6	**<0.01**	1.3	0.74
**Average Activity**	5.1	**0.02**	13.3	**<0.01**	23.3	**<0.01**			4.3	**0.04**	41.4	**<0.01**	3.1	0.37	5.8	0.12
**Median Activity (LMM)**	11.0	**<0.01**	1.5	0.22	0.6	0.90			1.0	0.32	2.4	0.50	1.5	0.69	2.8	0.43
$\dot{M}O_{2standard}$	132.1	**<0.01**	49.4	**<0.01**	100. 1	**<0.01**	123.9	**<0.01**	0.01	0.91	149	**<0.01**	51.6	**<0.01**	7.7	0.05
$\dot{M}O_{2average}$	95.1	**<0.01**	29.2	**<0.01**	31.0	**<0.01**	67.9	**<0.01**	0.3	0.56	114.4	**<0.01**	25.5	**<0.01**	2.9	0.42

Table S3. Summary of statistical outputs obtained from generalized linear models (GLM; family=Gamma, link=log) examining the effects of temperature (2°C or 8°C), prolonged starvation (90 days) and their interactions on energetic and morphological variables of adult brook char (Experiment 2). These analyses correspond with the data shown in Figures 4, 6, 7 and 8 in the main text. Significance ($p<0.05$) was assessed using type II Wald chi-square tests and is indicated in bold.

	**Temperature** (df=1)		**Feeding Status** (df=1)		**Lean Mass or Mass** (df=1)		**Temperature: Treatment** (df=1)	
	X^2	p	X^2	p	X^2	p	X^2	p
**Stomach Mass**	4.41	**0.036**	2.43	0.119	101.63	**<0.001**	0.05	0.819
**Pylorus Mass**	14.18	**<0.001**	131.79	**<0.001**	69.47	**<0.001**	0.145	0.704
**Intestine Mass**	19.52	**<0.001**	103.74	**<0.001**	145.45	**<0.001**	15.12	**<0.001**
**Relative Gut Mass**	19.22	**<0.001**	127.29	**<0.001**	136.46	**<0.001**	0.05	0.816
**Liver TG Content**	3.08	0.079	0.55	0.458			0.06	0.799
**Total Relative Liver TG**	8.28	**0.004**	4.79	**0.029**	1.77	0.183	0.01	0.936
**White Muscle TG Content**	2.44	0.118	46.23	**<0.001**			1.03	0.209
**Total Relative Carcass Protein**	2.63	0.105	1.59	0.208	21.12	**<0.001**	0.04	0.833
**Liver K_s**	10.77	**0.001**	0.04	0.846			0.29	0.593
**White Muscle K_s**	4.23	**0.040**	25.36	**<0.001**			0.16	0.685

TG, Triglyceride
K_s, Protein synthesis rate

Journal of Experimental Biology • Supplementary information

Table S4. Summary of statistical outputs obtained from linear models (LM) examining the effects of temperature (2°C or 8°C), prolonged starvation (90 days) and their interactions on energetic and morphological variables of adult brook char (Experiment 2). These analyses correspond with the data shown in Figures 3, 4, 6, 7 and 8 in the main text. Significance ($p<0.05$) was assessed using type II Wald chi-square tests and is indicated in bold.

	Temperature			Feeding Status			Lean Mass or log(Mass)			Temperature × Feeding Status		
	F	df	p	F	df	p	F	df	p	F	df	p
**Ventricle Mass**	9.29	1,45	**0.004**	4.53	1,45	**0.039**	155.33	1,45	**<0.001**	0.49	1,45	0.486
**Liver Mass**	20.49	1,45	**<0.001**	39.12	1,45	**<0.001**	95.59	1,45	**<0.001**	2.36	1,45	0.132
**Intestine Length**	0.86	1,45	0.360	45.34	1,45	**<0.001**	26.53	1,45	**<0.001**	1.76	1,45	0.192
**Gut:Heart Ratio**	2.49	1,45	0.122	87.86	1,45	**<0.001**	2.42	1,45	0.127	0.79	1,45	0.379
**Spleen Mass**	4.19	1,45	**0.047**	5.75	1,45	**0.021**	47.78	1,45	**<0.001**	0.72	1,45	0.382
**Body Fat**	0.03	1,46	0.868	48.41	1,46	**<0.001**				0.11	1,46	0.737
**Moisture Content**	0.35	1,46	0.555	104.09	1,46	**<0.001**				0.15	1,46	0.704
**Energy Density**	2.27	1,46	0.139	43.99	1,46	**<0.001**				2.28	1,46	0.138
**Gut TG Content**	0.788	1,46	0.379	62.21	1,46	**<0.001**				0.309	1,46	0.581
**Total Relative Gut TG Content**	2.77	1,45	0.103	117.13	1,45	**<0.001**	1.14	1,45	0.292	4.87	1,45	**0.032**
**Liver Protein Content**	3.83	1,45	0.057	0.01	1,45	0.925				1.63	1,45	0.209
**Total Relative Liver Protein**	37.75	1,44	**<0.001**	43.40	1,44	**<0.001**	1.19	1,44	0.281	1.46	1,44	0.234
**Gut Protein Content**	9.46	1,45	**0.004**	29.24	1,45	**<0.001**				7.03	1,45	**0.011**
**Total Relative Gut Protein**	0.61	1,44	0.438	0.86	1,44	0.358	6.45	1,44	**0.015**	6.91	1,44	**0.012**
**White Muscle Protein Content**	0.001	1,46	0.975	7.36	1,46	**0.009**				0.091	1,46	0.764
$\dot{M}O_{2standard}$	19.37	1,43	**<0.001**	107.69	1,43	**<0.001**	75.26	1,43	**<0.001**	1.57	1,43	0.218
$\dot{M}O_{2max}$	14.71	1,43	**<0.001**	0.09	1,43	0.764	46.22	1,43	**<0.001**	1.81	1,43	0.186
**Absolute Aerobic Scope**	12.18	1,43	**0.001**	0.25	1,43	0.620	36.69	1,43	**<0.001**	1.60	1,43	0.212
**Gut K_s**	4.23	1,44	**0.046**	35.38	1,44	**<0.001**				0.26	1,44	0.613
**Ventricle K_s**	51.96	1,44	**<0.001**	9.22	1,44	**0.004**				3.05	1,44	0.088
**Glucose**	1.39	1,46	0.245	10.19	1,46	**0.003**				0.01	1,46	0.930
**Hematocrit**	1.23	1,45	0.273	5.92	1,45	**0.019**				0.102	1,45	0.751
**Plasma Triglyceride**	0.74	1,36	0.397	6.63	1,36	**0.014**				3.60	1,36	0.066

TG, Triglyceride
$\dot{M}O_{2standard}$, Standard oxygen consumption
$\dot{M}O_{2max}$, Maximum oxygen consumption
K_s, Protein synthesis rate
